# Supplementary material for: Baseline Characterization of the Gut Microbiota of Field and Colony Populations of Phlebotomus tobbi and Preliminary Assessment of the Anti-Leishmanial Activity of Cultivable Bacteria
Source: Pathogens. 2026 Jun 23;15(7):658. doi: 10.3390/pathogens15070658 (PMC13416292; doi:10.3390/pathogens15070658)
Supplement: Supplementary file 1 [file pathogens-15-00658-s001.zip › pathogens-4357168-File S1/Composition_Barplots/Figures/charts/ApWkwBfkJnOWFjoAdNySzZP2BtSnGa_legend.pdf]

- k__Bacteria;p__Actinobacteria;c__Actinobacteria;o__Actinopolysporales;f__Actinopolysporaceae;g__Actinopolyspora
- k__Bacteria;p__Actinobacteria;c__Actinobacteria;o__Corynebacteriales;f__Corynebacteriaceae;g__Corynebacterium
- k__Bacteria;p__Actinobacteria;c__Actinobacteria;o__Corynebacteriales;f__Mycobacteriaceae;g__Mycobacterium
- k__Bacteria;p__Actinobacteria;c__Actinobacteria;o__Corynebacteriales;f__Nocardiaceae;g__Rhodococcus
- k__Bacteria;p__Actinobacteria;c__Actinobacteria;o__Micrococcales;f__Microbacteriaceae;g__Microbacterium
- k__Bacteria;p__Actinobacteria;c__Actinobacteria;o__Propionibacteriales;f__Propionibacteriaceae;g__Propionibacterium
- k__Bacteria;p__Actinobacteria;c__Actinobacteria;o__Pseudonocardiales;f__Pseudonocardiaceae;g__Saccharopolyspora
- k__Bacteria;p__Firmicutes;c__Bacilli;o__Bacillales;f__Staphylococcaceae;g__Staphylococcus
- k__Bacteria;p__Firmicutes;c__Bacilli;o__Lactobacillales;f__Enterococcaceae;g__Enterococcus
- k__Bacteria;p__Firmicutes;c__Bacilli;o__Lactobacillales;f__Leuconostocaceae;g__Fructobacillus
- k__Bacteria;p__Firmicutes;c__Clostridia;o__Clostridiales;f__Lachnospiraceae;g__Anaerostipes
- k__Bacteria;p__Firmicutes;c__Clostridia;o__Clostridiales;f__Lachnospiraceae;g__Blautia
- k__Bacteria;p__Firmicutes;c__Clostridia;o__Clostridiales;f__Ruminococcaceae;g__Faecalibacterium
- k__Bacteria;p__Proteobacteria;c__Alphaproteobacteria;o__Rhizobiales;f__Bradyrhizobiaceae;g__Afipia-Bradyrhizobium
- k__Bacteria;p__Proteobacteria;c__Alphaproteobacteria;o__Rhizobiales;f__Bradyrhizobiaceae;g__NA
- k__Bacteria;p__Proteobacteria;c__Alphaproteobacteria;o__Rhizobiales;f__Brucellaceae;g__Brucella-Ochrobactrum
- k__Bacteria;p__Proteobacteria;c__Alphaproteobacteria;o__Rhizobiales;f__Methylobacteriaceae;g__Methylobacterium
- k__Bacteria;p__Proteobacteria;c__Alphaproteobacteria;o__Rhizobiales;f__NA;g__Phreatobacter
- k__Bacteria;p__Proteobacteria;c__Alphaproteobacteria;o__Rhodospirillales;f__Acetobacteraceae;g__Asaia
- k__Bacteria;p__Proteobacteria;c__Alphaproteobacteria;o__Rickettsiales;f__Rickettsiaceae;g__Rickettsia
- k__Bacteria;p__Proteobacteria;c__Alphaproteobacteria;o__Sphingomonadales;f__Sphingomonadaceae;g__Sphingomonas
- k__Bacteria;p__Proteobacteria;c__Betaproteobacteria;o__Burkholderiales;f__Alcaligenaceae;g__Achromobacter
- k__Bacteria;p__Proteobacteria;c__Betaproteobacteria;o__Burkholderiales;f__Burkholderiaceae;g__Burkholderia-Paraburkholderia
- k__Bacteria;p__Proteobacteria;c__Betaproteobacteria;o__Burkholderiales;f__Burkholderiaceae;g__Ralstonia
- k__Bacteria;p__Proteobacteria;c__Betaproteobacteria;o__Burkholderiales;f__Comamonadaceae;g__Delftia
- k__Bacteria;p__Proteobacteria;c__Gammaproteobacteria;o__Enterobacteriales;f__Enterobacteriaceae;g__Enterobacter
- k__Bacteria;p__Proteobacteria;c__Gammaproteobacteria;o__Enterobacteriales;f__Enterobacteriaceae;g__Erwinia
- k__Bacteria;p__Proteobacteria;c__Gammaproteobacteria;o__Enterobacteriales;f__Enterobacteriaceae;g__Klebsiella
- k__Bacteria;p__Proteobacteria;c__Gammaproteobacteria;o__Enterobacteriales;f__Enterobacteriaceae;g__Morganella
- k__Bacteria;p__Proteobacteria;c__Gammaproteobacteria;o__Enterobacteriales;f__Enterobacteriaceae;g__Pantoea
- k__Bacteria;p__Proteobacteria;c__Gammaproteobacteria;o__Enterobacteriales;f__Enterobacteriaceae;g__Serratia
- k__Bacteria;p__Proteobacteria;c__Gammaproteobacteria;o__Oceanospirillales;f__Halomonadaceae;g__Zymobacter
- k__Bacteria;p__Proteobacteria;c__Gammaproteobacteria;o__Pseudomonadales;f__Moraxellaceae;g__Acinetobacter
- k__Bacteria;p__Proteobacteria;c__Gammaproteobacteria;o__Pseudomonadales;f__Pseudomonadaceae;g__Pseudomonas
- k__Bacteria;p__Proteobacteria;c__Gammaproteobacteria;o__Xanthomonadales;f__Xanthomonadaceae;g__Stenotrophomonas
